# Academic distress among undergraduate students during COVID-19: the relevance of SES and help-seeking behaviors

**DOI:** 10.3389/fpsyg.2023.1181009

**Published:** 2023-06-12

**Authors:** Liat Korn, Avi Zigdon, Nitza Davidovitch

**Affiliations:** ^1^Department of Health Systems Management, School of Health Sciences, Ariel University, Ariel, Israel; ^2^Department of Education Studies, Faculty of Social Sciences, Ariel University, Ariel, Israel

**Keywords:** academic distress, health indices, economic indices, undergraduate students, COVID-19

## Abstract

**Introduction:**

Academic distress has been frequently reported following the COVID-19 pandemic. This study estimates academic distress among undergraduate students, characterizes its nature in relation to economic, social, and health indicators, and examines the level of request for help following mental distress. Students with higher levels of academic distress were expected to show lower socio-economic status, lower social connections, and lower wellbeing indices.

**Methods:**

A cross-sectional study based on a structured anonymous questionnaire was delivered online to more than 1,400 undergraduate students from one university in Israel (women, 66.7%).

**Results:**

Academic distress was reported by 27.1% of the sample. Students who reported academic distress were more likely to report stress, negative psycho-somatic symptoms, changes in weight since COVID-19, low self-esteem, depressive symptoms, higher COVID-19 concerns, and higher security situation concerns. A hierarchic logistic regression model showed that the probability of reporting academic distress was 2.567 times higher (*p* < 0.001 95% CI [1.702, 3.871]) for those who reported lower family economic status before COVID-19 and 2.141 times higher (*p* = 0.004 95% CI [1.284, 3.572]) for those who highly reported depressive symptoms. In contrast, only 15.6% of those who reported academic distress sought help from academic authorities.

**Discussion:**

The significant associations of academic distress with health indices indicate that the self-reported distress was real and highly related to adverse health measures. A comprehensive, collaborative model that integrates psychological, economic, and social aspects of intervention is required in times of crisis within academic institutions.

## Introduction

In the early phase of the pandemic, the World Health Organization (WHO) noticed and alerted that the impact of COVID-19 would expand much more widely beyond the health sector (Samaan et al., 2021). Indeed, the pandemic significantly impacted society’s functioning and indirectly impacted academic teaching practices and students (Arima et al., 2020; Aristovnik et al., 2021).

Academic teaching methodologies needed to be updated (Sá and Serpa, 2020), and some had switched to alternative study methods (Pokhrel and Chhetri, 2021; Hadwin et al., 2022). These processes created uncertainty, technological concerns, distance from home, social isolation, anxieties, decreased family income, and concerns about future employment (Aristovnik et al., 2020). The emergency forced academic institutions to immediately identify the deficiencies in face-to-face student support services and provide them online (Bouchey et al., 2021). However, according to Lederer et al. (2021), students already had a high prevalence of mental health conditions, and as the COVID-19 pandemic spread, these risk factors and other health issues deteriorated. This had an impact on student’s grades and high levels of academic stress (Clark and Phillips, 2021; Lederer et al., 2021), which influenced the requirements for the continued support for their academic performance and mental health during the COVID-19 pandemic (Clark and Phillips, 2021; Prowse et al., 2021).

The pandemic also sharpened socio-economic disparities and accentuated digital inequality among students (Easterbrook et al., 2023). Demographic characteristics such as age, education, income, and ethnicity predict not only accessibility to “end-devices” and internet connection (to digital gaps in accessibility) but also translate to gaps in technological skills and the use of ICT (information and communications technology) on the internet (Mesch and Talmud, 2011). High socio-economic background was linked to some other matters concerning studying, including significant support from parents for educational aspects (Bol, 2020), high levels of self-motivation, ability to maintain studying and independence (Delevingne et al., 2020), and more hours for studying (Andrew et al., 2020). While all students may be affected by the consequences of the COVID-19 pandemic, students of lower socio-economic status have higher mental distress due to their limited financial capacity to obtain the necessary gadgets and internet connectivity (Alibudbud, 2021; Cleofas and Rocha, 2021).

As for health distress during the COVID-19 pandemic, research has shown that students in higher education settings were facing tremendous biopsychosocial stress (Hunt et al., 2021), mental distress, and depression (Romeo et al., 2021; Yu et al., 2021), with the mediating effect of lower self-esteem (Yu et al., 2021). Negative emotions could increase the frequency of health complaints and psycho-somatic symptoms (Zidkova et al., 2021). Studies have found that distress was also associated with eating as a coping method, which in turn was associated with increases in weight-promoting eating behaviors (Keenan et al., 2022). Food insecurity in undergraduates in Brazil was related to difficulties in maintaining weight and poor diet quality (Maciel et al., 2022). Lower sleep quality was found among United Kingdom undergraduates’ samples as a consequence of COVID-19 on mental health and wellbeing (Evans et al., 2021).

Students’ motivation is primarily related to their ability to create new friendships within academic settings (Davidovitch and Wadmany, 2021). Students who reported loneliness and difficulties in creating new social relationships also reported harm on their mental health and academic functioning and that they felt that their chances of persisting in the first year of their studies may have been harmed (Moeller and Seehuus, 2019; Thomas et al., 2020; Meishar-Tal, 2023). Social and emotional support reduces stress and the initial experience of loneliness in a new environment (Xerri et al., 2018). The COVID-19 closure period led to online learning from home, a situation that resulted in students not having any opportunities to meet face-to-face with their classmates (Stadtfeld et al., 2019). In a study conducted on medical students in Germany, first-year undergraduate students reported significantly higher levels of distress, anxiety, and depression than students during their second to fourth years of studies (Guse et al., 2021). Moreover, female students had higher odds of reporting high-risk acute stress compared to male students in Lebanon (Kassir et al., 2021) or depressive symptoms, anxiety, or insomnia among Italian university students (Amerio et al., 2021).

In Israel, just as in many other countries, the pandemic caught the academic systems by surprise. Around 320 thousand students in higher education needed to start digital online studying on the same day (Donitsa-Schmidt and Ramot, 2020). In March 2020, the first Israeli lockdown and restrictions were implemented all over the country. All higher education institutions immediately adopted remote online working and teaching and continued to work during the lockdowns (Nadiv, 2022). Jabbari et al. (2023) found that Israeli students who reported higher levels of social support were more emotionally available for learning. As suggested by the salutogenic theory, resistance to stress could be explained by a personal sense of coherence (SOC, Antinivskj, 1979). SOC demonstrates a dynamic feeling that the world is meaningful (perceiving life as meaningful and worthy of commitment and engagement), comprehensible (perceiving the world as structured and consistent), and manageable (perceiving one’s resources as adequate to manage an adversity) (Antonovsky, 1987). The purpose of this study was to estimate the rate of reported academic distress by university undergraduate students, to characterize its nature related to economic, social, and health indicators, and to examine the level of request for help following mental distress. Based on reviewed literature (Xerri et al., 2018; Stadtfeld et al., 2019; Guse et al., 2021; Romeo et al., 2021; Yu et al., 2021), we hypothesized that students with higher levels of academic distress were expected to show lower socio-economic status, lower social connections, and lower wellbeing indices.

## Materials and methods

This is a comprehensive cross-sectional study based on a structured anonymous questionnaire delivered online to undergraduate students in their classrooms in one university.

### Variables and instruments

The structured self-reported and anonymous questionnaire was based on an epidemiological design derived from two main sources: 1. Personal and Social Development Survey (Jessor et al., 2003); 2. Health Behaviors of School-aged Children–Israeli version of HBSC study questionnaire (Harel-Fisch et al., 2010; Pawar, 2020). Previous studies validated the questionnaire (Zukerman et al., 2016; Korn et al., 2021). The survey covered topics related to healthy psychological and social wellbeing, such as mental stress, self-image, psycho-somatic symptoms, academic studying, and security situation concerns. Several new questions were added regarding COVID-19 academic distress. To validate new questions in the questionnaire, the questionnaire was transferred to a pilot of 30 subjects, and the contents of the question items and their degree of relevance to what we intended to test were checked as well as the internal consistency between the items.

### Procedure

Ethical approval was obtained (AU-HEA-LK-20220220) from the Academic Institutional Ethical Review Board. After receiving approval from the academic institutional committee, contact was made with deans, departments heads, and lecturers using the lessons list from each studying faculty’s secretariat. With permission from the lecturer, surveyors entered more than 100 classrooms in the 10 last minutes of the lessons from March to June 2022 within one university. The surveyors presented the questionnaire through a link to those who were willing to participate in the study. The average response rate (83.4%) for all the classes was calculated after subtracting those who refused to participate from those presented in the classrooms. The questionnaires were delivered online inside the classroom by a link copied to the students’ mobile phones or laptops. Those who did not have electronic devices could use a printed version or tablets that were brought to the classrooms by the surveyors. Data were collected using the Qualtrics platform. After removing participants due to missing values or too short a response time, the file of the final sample included 1,410 participants.

### Sample description

The survey was delivered in one university in Israel from April to June 2022 at the end of the second year of the COVID-19 pandemic. Many lessons were still being delivered online and did not require in-person presentation. This needs to be considered for sample representation. The sample represented all undergraduates’ students in each faculty presented in classrooms. The unweighted sample contained 941 women (66.7%) and 469 men (33.3%). As shown in Table 1, the weighted file contained 51.3% women and 48.7% men. The mean age was 25.7 (SD- 4.1), most of the sample (63.4%) comprised single people, and 82.3% of the sample had an average or above-average family socio-economic status.

**Table 1:** Characteristics of undergraduate students who reported health or economic distress that interfered with academic performance (%; weighted for gender).

Topic	Variables	Values	Distress % *M* (SD)	No distress % *M* (SD)	*Sig.	Total %
Socio-demographic	Gender	Woman	54.0	50.2	NS	51.3
		Man	46.0	49.8		48.7
	Age	Mean (SD)	25.7 (4.1)	25.4 (5.0)	NS	36.4
	Family status	Married	39.8	35.1	NS	63.6
		Single	60.2	64.9		
	Family SES	Average and above average	**70.1**	**89.6**	***p* < 0.001**	82.3
		Below average	**29.9**	**13.1**		17.7
	Family SES after COVID-19	Same and better	**65.5**	**83.8**	***p* < 0.001**	78.8
		Worse	**34.5**	**16.2**		21.2
Academic	Faculty of studying	Health sciences	36.1	33.3	NS	34.1
		Nature sciences	11.8	13.4		12.9
		Social sciences	22.6	22.3		22.3
		Engineering	22.6	21.9		22.1
		Medicine	3.5	4.8		4.5
		Communication	3.5	4.3		4.1
	Year of study	First	**41.6**	**47.0**	**0.030**	45.6
		Second	**34.9**	**34.6**		34.7
		Third	**20.5**	**17.2**		18.1
	Grades mean	Mean (SD)	**7.3 (1.85)**	**7.5 (1.53)**	**0.019**	
Social	Close friends	No friends	**12.6**	**7.6**	**0.009**	8.9
		At least one friend	**87.4**	**92.4**		91.1
	Connection with friends while learning remotely	Felt connected	29.6	32.6	NS	31.8
		Not very connected	29.6	31.7		31.1
		Felt disconnected	40.8	35.7		37.1
	Connection with friends today	Felt connected	**72.9**	**84.5**	***p* < 0.001**	81.4
		Not very connected	**17.6**	**10.0**		12.1
		Felt disconnected	**9.5**	**5.4**		6.5
Wellbeing	Stress scale	Mean (SD)	**2.6 (0.7)**	**2.2 (0.6)**	***p* < 0.001**	
	PSS	Mean (SD)	**2.8 (0.9)**	**2.2 (0.8)**	***p* < 0.001**	
	Changes in weight since COVID-19	No change	**32.9**	**48.3**	***p* < 0.001**	44.1
		Gained weight	**46.8**	**37.3**		39.9
		Lost weight	**20.3**	**14.4**		16.0
	Changes in sleeping habits since COVID-19	No change	**55.1**	**74.0**	***p* < 0.001**	68.9
		Yes, for better	**8.6**	**4.2**		5.4
		Yes, for worst	**36.2**	**21.8**		25.7
	Low self-image	Mean (SD)	**2.1 (0.5)**	**1.9 (0.4)**	***p* < 0.001**	
	Depressive symptoms	Mean (SD)	**1.5 (0.5)**	**1.2 (0.3)**	***p* < 0.001**	
	COVID-19 concerns scale	Mean (SD) higher = less concerns	**3.0 (1.1)**	**3.5 (1.1)**	***p* < 0.001**	
	Security situation concerns	Mean (SD)	**2.3 (0.8)**	**2.1 (0.7)**	***p* < 0.001**	

Significant values were bolded. M, Mean; SD, Standard deviation; SES, Socio-economic status; PSS, Psycho-somatic symptoms; *Sig, Significant results from chi-square/*t*-test.

### Variables description

#### Dependent variables

Academic distress was presented to the participants using five questions, the first three of which were: 1. *Perceived distress*–the participants were asked as follows: “During the last 2 years, have you felt a health or financial hardship that interfered with your academic performance?” (1. Yes, 2. No). 2. S*haring with university authorities*–who among the university authorities the participants chose to share with. The responses were: 1. Administrative staff in the department; 2. Academic staff in the department; 3. Dean of Students; 4. Others. 3. *Rate sharing*–the participants were asked to rate how well the support they received from the university authorities was. The responses were: 1. Very good, 2. Good, 3. Not good enough, 4. Very bad.

### Socio-demographic measures

The participants were asked for their *gender* (man, woman), for their *age,* which was calculated the from year of birth, and for their *family status* 0. married/in relations; 1. single (bachelor/divorced/separated/widower).

### Academic studies

The participants were asked which *faculty of studying* they were studying in (health sciences, social sciences, engineering sciences, nature sciences, medicine, or communication) and their *year of study* (First, second, or third).

### Academic achievement

The participants were asked to describe their grade point average in the last semester. The responses ranged from failure–50 to excellence–100 as follows: 1. 50–54; 2. 55–59; 3. 60–64; 4. 65–69; 5. 70–74; 6. 75–79; 7. 80–84; 8. 85–89; 9. 90–94; 10. 95–100.

### Socio-economic status measures

The participants were asked two questions regarding their economic status. 1. *Family economic status*–“What is the average monthly income of your family?.” The responses were: 1. Much above average, 2. Above average, 3. Average, 4. Below average, 5. Much below average. 2. *COVID-19 changed economic status*–the participants were asked for their opinion on the financial situation of their family today compared to the situation before the outbreak of the COVID-19 pandemic. The responses were: 1. The situation today is much worse, 2. The situation today is less good, 3. The situation has not changed, 4. The situation is better today, 5. The situation today is much better.

### Social connectedness with friends

The participants were asked about the *number of their close friends* at the university. The responses were: 1. None, 2. One, 3. Two or three, 4. Four or more. Two more questions were related to the *connection with friends while learning remotely*–“To what extent did you feel connected or disconnected from your classmates during the distance learning period?” and *connection with friends today*–“Today, after returning to campus, how connected or disconnected do you feel from your friends?.” The responses were: 1. Feel very connected, 2. Feel connected, 3. Not so connected, 4. Feel a little disconnected, 5. Feel very disconnected.

### Wellbeing measures

*Stress scale*–four items measured stress in the last month: Academic, residential, family life, and stress from personal or social life (Cronbach’s alpha 0.60; Jessor et al., 2003; 0.72 in the current study). *Psycho-somatic symptoms*–eight psycho-somatic symptoms (headache, abdominal pain, back pain, bad mood–grumpy/depressed, anger, nervousness, difficulty falling asleep, dizziness) originating from the Health Behavior in School-aged Children (HBSC) self-report scale (Ravens-Sieberer et al., 2008; Zukerman et al., 2016). *Changes in weight since COVID-19*–the participants were asked if they thought there had been a change in their weight since the outbreak of the COVID-19 crisis. The responses were: 1. There was no change in my weight, 2. Yes, I gained weight, 3. Yes, I lost weight. *Changes in sleeping habits*–the participants were asked if there had been a change in their sleeping habits since the outbreak of the COVID-19 crisis. The responses were: 1. There was no change, 2. Yes, there was a change for the better, 3. Yes, there was a change for the worse. *Self-esteem*–seven items for measuring self-esteem were combined: get along with others, make decisions about important life issues, do well in school, perceive oneself as attractive and appealing, deal with setbacks and disappointment, be physically attractive, and feel satisfied with oneself (Cronbach’s alpha was 0.78; Jessor et al., 2003). For these items, higher scores represent lower self-esteem. *Depressive symptoms*—four items measured cognitive, emotional, physiological, and motivational aspects of depressive symptoms originating from the Back Depression Inventory (BDI-II) self-report measure (Beck et al., 1996). *COVID-19 concerns scale*–mean score of three variables: concerns regarding the effects of the COVID-19 pandemic on academic achievements, on their social status, and on their economic situation (Cronbach’s alpha was 0.81). *Security situation concerns*–mean score of eight items on a scale from 1. not at all to 5. very much (Cronbach’s alpha was 0.80). One item was reversed, and another was removed due to its lower reliability with other items.

### Data analysis

Using IBM statistics SPSS-28, we started applying weighting analysis for gender to match a gender ratio of 50–50. Reliability tests and factor analyzes were applied for creating scales for stress, psycho-somatic symptoms, self-image, depressive symptoms, COVID-19 concerns, and security situation concerns. Following that, chi-square analyzes with cross-tabulation frequency and t-test were conducted to determine differences between independent groups (Table 1) as well with descriptive frequency (Table 2). Logistic regression was performed in three steps for the association between academic distress and social and wellbeing variables (Table 3). The normal distribution of outcome variables was assessed in accordance with Kim (2013).

**Table 2:** Frequency distribution of students reporting of informing university authorities.

	Variables	Values	*n*	%
Informed university authorities	University authority’s participants chose to inform of their academic distress	Department administrative staff	13	27.6
		Department academic staff	15	31.9
		Student’s dean	10	21.2
		Others	9	19.1
Degree level support	Participant’s degree level of support they received from the university authorities	Very good	6	14.6
		Good	11	26.8
		Not good enough	10	24.4
		Very bad	14	34.1

**Table 3:** Outcomes from hierarchical three-steps logistic regression for association between academic distress and social and wellbeing variables.

Steps	Variables		OR	*P*	95% CI		*R*^2^ (*N*)
					Lower	Upper	
1	Socio-demographic	Gender (1 = Man)	0.861	0.303	0.647	1.145	0.089 (1125)
		Age	1.013	0.389	0.984	1.042	
	Family SES	Before COVID-19 (1 = Below average)	**2.508**	*p* < 0.001	1.793	3.508	
		After COVID-19 (1 = Worse)	**2.460**	*p* < 0.001	1.793	3.376	
2	Family SES	Before COVID-19 (1 = Below average)	**2.427**	*p* < 0.001	1.726	3.412	0.101^a^ (1101)
		After COVID-19 (1 = Worse)	**2.542**	*p* < 0.001	1.846	3.500	
	Academic	Year of study (1 = first year)	0.798	0.131	0.595	1.070	
		Grades mean	0.918	0.053	0.842	1.001	
3	Family SES	Before COVID-19 (1 = Below average)	**2.567**	*p* < 0.001	1.702	3.871	0.274^a^ (934)
		After COVID-19 (1 = Worse)	**1.735**	0.005	1.177	2.558	
	Academic	Grades mean	1.735	0.309	0.950	1.177	
	Social	Number of close friends where studying	1.056	0.620	0.852	1.309	
		Connection with friends today	1.156	0.202	0.925	1.443	
	Wellbeing	Stress scale	**1.642**	*p* < 0.001	1.220	2.211	
		Psycho-somatic symptoms	**1.545**	*p* < 0.001	1.200	1.989	
		Change in weight since COVID-19	**1.448**	0.043	1.012	2.072	
		Changes in sleeping habits since COVID-19	1.382	0.088	0.953	2.004	
		Low self-esteem scale	0.939	0.794	0.585	1.508	
		Depressive symptoms scale	**2.141**	0.004	1.284	3.572	
		COVID-19 concerns scale	0.867	0.099	0.731	1.027	
		Security situation concerns scale	0.962	0.748	0.759	1.220	

^a^Standardized for age and gender. Significant values were bolded. M, Mean; SD, Standard deviation; SES, Socio-economic status.

## Results

Students were asked if during the last 2 years they felt economic or health distress that interfered with their academic achievement. Among a large sample of more than 1,400 undergraduates, 301 (27.1%) reported distress. Table 1 presents their socio-demographic, economic, academic, and wellbeing characteristics in frequencies with chi-square significance for determining differences between independent groups. Mean and standard deviation were presented as scale variables. The findings show differences between students according to their reports of academic distress in five groups of variables. Reports of academic distress did not vary according to gender, age, or family status. Regarding academic variables, the students’ grades mean was significantly lower among those who reported academic distress (*M* = 7.3, SD = 1.85) than those who did not report of distress (*M* = 7.5, SD = 1.53). Significant differences were found related to SES. Students who reported a family economic status below average, especially after the COVID-19 pandemic, were more likely to report academic distress. All variables regarding emotional and physical wellbeing show that the students who reported academic distress were more likely to report worse emotional and physical wellbeing conditions: they highly reported stress, negative psycho-somatic symptoms, changes in weight since the COVID-19 pandemic, low self-esteem, depressive symptoms, higher COVID-19 concerns, and higher security situation concerns.

The findings in Table 3 show the outcomes from the hierarchical logistic regression analysis in three steps for detecting associations between academic distress and the study variables (Step 1–Socio-demographic and family economic status; Step 2–Socio-demographic, family economic status, and academic; Step 3–Socio-demographic, economic status, social, and wellbeing).

The final model in step 3 shows, with 27.4% explained variance, that SES and wellbeing characteristics–stress, psycho-somatic symptoms, change in weight since the COVID-19 pandemic, and depressive symptoms–were significant factors associated with academic distress. The probability of reporting academic distress was 2.567 times higher [*p* < 0.001 95% CI (1.702, 3.871)] for those who reported lower family SES before the COVID-19 pandemic and 1.735 times higher [*p* < 0.001 95% CI (1.177, 2.558)] for those who reported worse family SES after COVID-19. The probability of reporting academic distress was 1.642 times higher [*p* < 0.001 95% CI (1.220, 2.211)] for those who reported higher stress levels, 1.545 times higher [*p* < 0.001 95% CI (1.200, 1.989)] for those who reported higher psycho-somatic symptoms, 1.448 times higher [*p* = 0.043 95% CI (1.012, 2.072)] for those who reported instability in weight change, and 2.141 times higher [*p* = 0.004 95% CI (1.284, 3.572)] for those who highly reported depressive symptoms.

Only 15.6% of those who reported academic distress sought help. Table 2 presents a description of the frequency distribution of student reports informing the university authorities. A total of 47 students chose to inform one of the university authorities–the mode was the department academic staff (*n* = 15, percent = 31.9%). The degree level of support the participants received from the university authorities was mostly not positive (*n* = 24, percent = 58.5%).

## Discussion

This paper examined academic distress 2 years after the COVID-19 pandemic lockdown in a large sample of undergraduate university students and characterized its economic, social, and health nature and the level of request for help. Students with higher levels of academic distress were expected to show lower socio-economic status, lower social connections, lower wellbeing indices, and more requests for help. More than a quarter of the sample (27.1%) reported academic distress. As well described in former studies, studying during the pandemic required updated tools (Sá and Serpa, 2020; Pokhrel and Chhetri, 2021) and was accompanied by uncertainty, technological concerns, distance from home, social isolation, anxieties, and income concerns (Aristovnik et al., 2020). Not only that, but academic stress may have impacted students’ grades (Clark and Phillips, 2021; Lederer et al., 2021), as also found in this study. Economic inequality in academic learning can have effects on the characteristics of future students and on their ability to succeed.

As expected, this paper’s findings demonstrated significant differences related to SES. Students who reported family economic status below average, especially after the COVID-19 pandemic, were more likely to report academic distress. With the background of the reviewed studies (Mesch and Talmud, 2011; Andrew et al., 2020; Bol, 2020; Delevingne et al., 2020; Alibudbud, 2021; Cleofas and Rocha, 2021), it is clear how lower SES might have had a negative effect on studying and academic achievements during the pandemic. Moreover, economic status impacted other aspects of life socially, emotionally, and physically. Our findings showed that students who reported academic distress were more likely to report stress, negative psycho-somatic symptoms, changes in weight, low self-esteem, depressive symptoms, higher COVID-19 concerns, and higher security situation concerns, as supported by the literature published after the COVID-19 outbreak (Evans et al., 2021; Hunt et al., 2021; Romeo et al., 2021; Yu et al., 2021; Zidkova et al., 2021; Keenan et al., 2022; Maciel et al., 2022). All these strong significant associations found between academic distress and health indices indicate that the self-reported distress was real and highly connected to negative health measures. The regression analysis also demonstrated the importance of economic and wellbeing measures to academic distress. The strongest predictors for academic distress in the final model were below-average family SES before the COVID-19 pandemic (2.5 times higher) and depressive symptoms (2.1 times higher).

The study findings also showed that the reporting of academic distress during the 2 years after the COVID-19 outbreak was similar among genders, different ages, different marital statuses, and those studying in different faculties. Different groups of these students reported academic distress after the COVID-19 outbreak in a similar manner. In contrast, the reviewed literature showed higher odds of reporting stress (Kassir et al., 2021) or depressive symptoms, anxiety, or insomnia (Amerio et al., 2021) among women students. A possible explanation for the discrepancies between the findings and the existing literature could be due to the timing of the study in relation to the pandemic. Two years after the pandemic, reports of distress can be expressed differently compared to the time of the lockdowns or immediately after. Beyond that, there could be variation in the severity of distress that might affect the reports. The feeling of distress was widespread among students with different characteristics. Academic institutions that initiate social activities for students increase the chance that their students will develop social relationships and have a rich and satisfying student experience (Alharthi, 2019), which can have a positive effect in reducing the feeling of distress. As suggested by Antonovsky (1987) and Antinivskj (1979), a lower personal sense of coherence will lower the possibility of handling distress. If a person perceives less meaningfulness of the world during a pandemic, and world inconsistency and no resources to manage their feelings, distress will take over. At the same time, despite the rates of academic distress, the relative share of those who sought help was low: only 47 students out of the 301 students who reported distress (15.6%) sought help, and the majority negatively rated the help they had received. Academic institutions that need to respond to a new problem are supposed to take care of students’ needs and adapt to changes. The department, faculty, and dean should provide a solution and service for students in times of need. The administrative system, in order to comply with student’s needs, must use expert powers from health and mental support systems to find the right response. Teaching-Promotion units should strengthen learning outputs, social–emotional learning, and working in teams. The pandemic has greatly sharpened this need.

To improve students’ social cohesion during crisis, it is recommended to locate tools such as digitization learning groups. Social cohesion should be accomplished through tools other than those employed in regular times and in combination with social gatherings that enable students to study together in small groups. It is problematic to portray seeking help at one’s place of higher education, the place that largely determines one’s professional future and where one is examined and has their progress checked, as needing help. This matter puts the student in a problematic place. People may have chosen to not seek help because of stigma, and the academic institution may not have necessarily encouraged it. The appeal may not have been given sufficient legitimacy. The university as a higher education institution should be relevant in dealing and caring about the mental distress of its students. It is also possible that students experienced the normalization of distress and that they perceived their distress as similar to others and did not necessarily want to complain or ask for help from their institution. Defensiveness became common during the pandemic, which was captured as an emergency situation in which healthy people died. The pressures of students were beyond normal. At this time, there should be an integration between the psychological, economic, and social sides that the academic institution should provide for its students. It is required to create a comprehensive collaborative model in times of crisis, linking the administrative and academic bodies, between the departments and between the faculty members. The COVID-19 pandemic has shown that the dean must work in a disciplinary model in a world of changes and a world of Zoom. To fulfill students’ needs, a combination of financial counseling with psychologists who support stress might be more sufficient for students in a time of crisis. A body that does not know how to correspond and respond in times of crisis should not continue to hold students. If academic institutions do not know how to respond to people in real time, they will lose them. It is possible that staff may improve in the future with more training, peer support groups, and self-care practices. The global pandemic presented unprecedented challenges, and even university staff faced personal losses while simultaneously shouldering the professional responsibility of supporting students.

### Limitations and future research

This study is not without limitations. First, the sample was based on students of one university, which decreases the chance that this sample properly represents all students in all Israeli universities. In addition, as stated in the Methods section, the ratio of women-to-men participants was biased toward women. Although the sample was diverse and was carried out on multiple university departments, the sampling bias must still be taken into account. Second, self-report studies can influence research results. The timing of the delivery of the questionnaires, the circumstances of the participants while filling out the questionnaire, and the time during the academic year, among other factors, can affect the perception of academic pressure. Third, this study did not investigate the services that the university provided to its students during the COVID-19 pandemic in order to match the student reports, nor did it elaborate on students’ barriers for seeking help. Therefore, further research should address the dynamics of information collection in this type of questionnaire and examine the dynamics of the relationships between the organizational systems at the university and the students’ reporting of distress.

## Data availability statement

The datasets presented in this article are not readily available because requires ethics committee approval. Requests to access the datasets should be directed to Liatk@ariel.ac.il.

## Ethics statement

The studies involving human participants were reviewed and approved by the research ethics committee of the Ariel University (ref AU-HEA-LK-20220220) reviewed and approved all aspects of this study. The patients/participants provided their written informed consent to participate in this study.

## Author contributions

LK: original draft of the manuscript, formal analysis, and data curation. LK, ND, and AZ: conceptualization and writing review, editing and visualization. LK and AZ: methodology, investigation, and validation. All authors contributed to the conception of the research idea. All authors contributed to the article and approved the submitted version.

## Conflict of interest

The authors declare that the research was conducted in the absence of any commercial or financial relationships that could be construed as a potential conflict of interest.

## Publisher’s note

All claims expressed in this article are solely those of the authors and do not necessarily represent those of their affiliated organizations, or those of the publisher, the editors and the reviewers. Any product that may be evaluated in this article, or claim that may be made by its manufacturer, is not guaranteed or endorsed by the publisher.
